# Cross-sectional study of prevalence of dementia, behavioural symptoms, mobility, pain and other health parameters in nursing homes in Austria and the Czech Republic: results from the DEMDATA project

**DOI:** 10.1186/s12877-018-0870-8

**Published:** 2018-08-13

**Authors:** Stefanie R. Auer, Margit Höfler, Elisabeth Linsmayer, Anna Beránková, Doris Prieschl, Paulina Ratajczak, Michal Šteffl, Iva Holmerová

**Affiliations:** 1 Danube University Krems, Dr.Karl-Dorrekstrasse 30, 3500 Krems, Austria; 2 Charles University, Šimůnkova 1600, 8- Kobylisy, 182 00 Prague, Czech Republic; 3 MAS Alzheimerhilfe, Lindau Strasse 28, 4820 Bad Ischl, Austria

**Keywords:** Nursing home, Dementia prevalence, Prevalence of behavioural symptoms, Pain, Malnutrition

## Abstract

**Background:**

This paper provides a first comparative exploratory analysis of our findings from DEMDATA, a collaborative project between Austria and the Czech Republic. Analysed here are data from the residents and the environment assessment protocol.

**Methods:**

In a cross sectional study design, residents from randomly drawn and stratified nursing homes were investigated using a common study protocol.

**Results:**

From a total resident pool of 1666 persons, 1085 (571 in Austria, 514 in the Czech Republic) persons signed a consent form and participated in the data collection.

More than 70% of residents assessed were female and the population was on average 85 years old. A discrepancy between the presence of a medical diagnosis in the charts of the residents and the results of cognitive testing was found. In Austria, 85.2%, in the Czech Republic 53.0% of residents had cognitive impairment. In Austria 80.0%, and in the Czech Republic 56.7% had behavioural problems. With respect to pain, 44.8% in Austria, and 51.5% in the Czech Republic had mild to severe pain. 78.4% of Austrian and 74.5% of the residents had problems with mobility and both populations were in danger of malnutrition.

**Conclusions:**

Most of the prevalence rates are comparable with previous studies also using direct resident assessment. Variations in prevalence rates seem to result mainly from the assessment technique (direct cognitive testing vs. medical chart review). The high prevalence rates for dementia, behavioural symptoms, pain and malnutrition indicate an immediate call for attention to further research and practice development.

## Background

According to Alzheimer’s Disease International, about 46.8 million persons suffer from dementia worldwide [1]. This number is expected to increase to 74.7 million persons by 2030 and to 131.5 million by 2050. Because the symptoms of dementia have been shown to be one of the most important factors associated with institutional long-term care (iLTC) admission [2], providing guidelines and concepts for high-quality iLTC for persons with dementia in institutions (such as nursing homes) is a particular challenge for most countries worldwide. However, the development of such guidelines or innovative concepts of care is often hindered as there is a lack of reliable information on the cognitive, physical and social status of persons living in iLTC. For instance, the OECD (Organization for Economic Co-operation and Development, [3]) as well as the European Commission (EC [4]) have repeatedly pointed out that the progress in dementia care and long-term care research is slow and more research data are needed. Moreover, several authors point to the necessity of international collaborative studies as a possibility to hasten progress [5, 6].

Studies are still few in Europe providing solid basic data on prevalence and severity of dementia, functioning and behavioural problems for iLTC, and even if these data exist, they often origin from institutions within one country or within a single geographical region. Although the findings of each of these studies are without doubt valuable, it is nevertheless difficult to directly compare findings from different studies and/or countries or to apply guidelines in one country based on such findings to another country. This might be because of methodical differences (e.g., different approaches to assess dementia) [7] but also because of differences in the underlying concepts and definitions of “nursing home” [8]. Moreover, most of the studies that compare dementia or dementia-related factors across countries rely on a post-hoc analysis of existing data. For instance, Testad and colleagues investigated the relationship between agitation and neuroleptic drug use across three different countries. To this end, they compared post-hoc baseline data from three different intervention studies on agitation in residents in Austrian, UK and Norwegian nursing homes [9]. In another study, the structure of long-term care and nursing homes in 10 European countries was compared by analysing government documents, statistical yearbooks and journal articles [10]. In the SHELTER study [6], an international database was founded providing basic epidemiologic data and prevalence rates. However, this study used convenient samples and the results cannot be generalized.

Based on different European studies, the prevalence of persons with dementia living in nursing homes is estimated to be about 60–80% [11, 12]. This high variation is mainly caused by the methodological differences of the studies which again makes it difficult to directly compare the findings. Whereas some studies determine the frequency of dementia via chart review or ratings by nursing staff [5], other studies directly screen the residents’ cognitive abilities [11]. When comparing the prevalence of dementia based on chart review and direct assessment, discrepancies are observed. In a study by Lithgow and colleagues 58% of residents in Glasgow nursing homes were found to have a medical dementia diagnosis in their resident charts. Further 31.8% showed a possible dementia based on clinical tests, leading to a prevalence rate of 89.8% in total [11]. Likewise, another study identified 83.8% of participants with dementia at the time of admission to the nursing home although for only 55.9% a dementia diagnosis was documented in the records of the residents [12]. Consequently, there seems to be a discrepancy between the number of people in nursing homes with and without diagnosed dementia which is alarming as it directly indicates that a substantial fraction of persons living in nursing homes do not receive the special care needed [13]. With regard to pain and neuropsychiatric/behavioural symptoms prevalence rates of 47.9% and 92.0% were reported respectively [5, 14, 15].

Even if there are studies on the prevalence of dementia, behavioural symptoms, and pain across Europe, this respective data is missing for Austria as well as for the Czech Republic. In 2016, the project DEMDATA was therefore started in order to provide such basic data for persons living in Austrian and Czech nursing homes. In particular, data on cognition, functioning, behavioural symptoms, pain, quality of life and further health parameters (e.g., number of falls, hospital stays) with regard to the residents and the environmental factors (e.g., size of institution, number of staff) were collected. Also the situation of the care team and the relatives was assessed.

The aim of the current paper is to provide first findings from this DEMDATA project with regard to the environmental factors (e.g., number of rooms, facilities, staff ratio) and residents’ data on cognition, functioning, behavioural symptoms, pain, quality of life and further health parameters. To this end, only the instruments applying to residents and environmental factors from the study protocol were analysed although the protocol contains additional variables for relatives, care team and qualitative variables which will be analysed in subsequent publications [16].

The following research questions were addressed in the current analysis:

- What is the prevalence of dementia, cognitive impairment and different health parameters (such behavioural problems, pain and functioning) in Austrian nursing homes and in nursing homes in the Czech Republic?
- Do these prevalence rates differ between the two countries?

## Methods

### Study design

In the DEMDATA project, a cross-sectional mixed-methods design was used in order to assess four domains of parameters related to (1) residents, (2) care team, (3) relatives and (4) environmental features. A common study protocol and assessment methodology was developed and a common data entry system was organized [16]. For the purpose of the current paper, we focus on the presentation and analysis of data from the DEMDATA resident and environmental features protocol only.

### Study population selection

In the literature, prevalence rates between 60 and 80% are reported [11, 12]. This information was used for the sample size calculation. We applied the formula for sample size calculation in descriptive studies for proportions [17, 18]. Using a 60% prevalence rate, a precision value of 0.05 and a *Z*-value of 1.96, the formula yielded a minimum sample size of 369 persons per site (country). Both study sites agreed to include a minimum of 500 residents per country into the study. In order to minimize travel expenses, only nursing homes of the federal states of Upper Austria (Austria) and Central Bohemia and Prague (Czech Republic), respectively, were considered. In Austria, 16 sites were initially randomly selected and stratified according to organizational features (50% state owned, 25% municipal, and 25% private) from all 128 available Upper Austrian nursing homes. Eight of these 16 nursing homes (five state owned, two municipal, one private) agreed to take part in the study. In the Czech Republic 159 nursing homes are registered in Central Bohemia and the Prague region and 14 nursing homes (seven municipal, five non-profit, and two for-profit nursing homes) participated in the study.

In the randomly selected study environments of both countries, all residents were given the same chance to participate in the study. However, in some nursing homes of the Czech Republic, only some wards were made accessible to the researchers. All participants had to give their informed consent (in case of the inability to give written consent, a legal representative had to provide consent). Participants were either permanently living in the selected nursing home or in respite care (independent of their length of stay). Excluded from the study were only persons with an acute serious health crisis (i.e. intensive care) or persons in the process of dying. Even if there was a written consent by a relative, in case of verbal or nonverbal decline of consent by the resident, the direct testing was not performed. Testers were well trained in communicating with elderly persons and persons with dementia of all stages and the testers created a supportive and warm atmosphere during the testing procedure. The study protocol and study methodology was approved by ethic committees in Austria and the Czech Republic.

### Data collection

Sociodemographic data and medical diagnoses were gained via chart review. All psychological assessments were conducted by a clinical psychologist or, as was the case in the Czech Republic, by a trained evaluator from the Czech Alzheimer Society who has experience in the work with persons with dementia. The clinical psychologists and evaluators were familiar with the diagnostic criteria for dementia. Dementia was defined according to the DSM V criteria of the American Psychiatric Association [19] as a) a significant cognitive decline from a previous level of performance in one or more cognitive domains (complex attention, executive function, memory, learning); b) interfering of cognitive deficits with independence in everyday activities; c) cognitive deficits being not due to a delirium or due to another mental disorder. In the Czech Republic, evaluators were additionally supervised by a Geriatrician (I.H., senior author of this publication). In Austria, all non-direct tested information (e.g. nutritional status, activities of daily living (ADL) status, number of falls and hospital stays and all environmental factors such as room facilities, social activities etc.) was collected by a research assistant, recruited from the nursing home care team for the duration of the data collection in the respective nursing home. Interviews with the nursing home administrator were performed by the study coordinators in the respective countries. Data were collected between September 2016 and June 2017 (Austria) and between August 2016 to August 2017 (Czech Republic), respectively. The data entry was performed by the testers and the study coordinators and data were entered into a common study data base located at the Danube University in Austria.

### Test instruments used

In order to assess the severity of dementia, the Global Deterioration Scale (GDS) scale was used [20]. The GDS is a 7-point Global Scale taking the severity of cognitive, functional and behavioural symptoms of pre-dementia and dementia stages into account. The GDS has satisfactory scale quality criteria (for example correlation with the German MMSE (validity): *r* = .86, [21]; reliability: *r* = .92; [22]). Within the GDS, each stage is numbered (1–7); stages 1–3 represent pre-dementia stages; stages 4–7 dementia stages. The GDS staging procedure is supported by the assessment of behavioural symptoms, functional symptoms and cognitive symptoms. The cognitive status was assessed using the Mini Mental State Examination (MMSE; [23]). The MMSE is a brief, standardized and widely used method which assesses orientation, attention, immediate and short-term recall, language, and the ability of persons to follow simple verbal and written commands (reliability: *r* = .83, validity: *r* = .78; [23]). The score of the MMSE ranges from 0 to 30 with a higher score reflecting a better cognitive functioning. Concentration, short-term memory, long-term memory and orientation were assessed with the Brief Cognitive Rating Scale (BCRS, [24]). This is a 7-point scale (interrater reliability .85–.97; [25], validity: *r* = .9; [26, 27, 24]) corresponding to the seven stages of the GDS. In addition, the Clock drawing test [28] was used as a screening test for cognitive impairment with a score from 1 (no impairment) to 6 (high impairment; validity: *r* = .65; [25], reliability: *r* = .95; [29]).

The presence of behavioural symptoms was assessed with the BEHAVE-AD-FW [30]. This scale assesses seven domains of behavioural pathology in Alzheimer’s disease (i.e., delusions, hallucinations, activity disturbances, aggressiveness, diurnal rhythm disturbances, affective disturbances, anxieties and phobias) as reported by a member of the care team. It also contains a global rating of symptom severity (ICCs .69 to .98 for the seven symptom categories, ICC for the total score: .9; [30]). In addition, the Empirical Behavioural Pathology in Alzheimer’s Disease Assessment Scale (E-BEHAVE-AD, [31]) was used as a direct observational version of the BEHAVE-AD-FW. It assesses behavioural pathology in AD using the same categories as the BEHAVE-AD-FW, additionally taking the perspective of the person with dementia into account. The E-BEHAVE-AD is filled in on the basis of a direct interview with the resident (ICC = .97; [31]).

Functioning was assessed via the Functional Assessment Staging of Alzheimer Disease (FAST, [32]; validity: 0.83 to 0.94 [28]; ICC: .86; [33]) and the KATZ Index of Independence in Activities of Daily Living [34]. The Katz Index has been found to be both internally consistent and strongly associated with quality of life measures [35]. Mobility was assessed via the Timed-get-up-and-go Test which demonstrated a good inter- and intrarater reliability (both ICC’s .99) and a good content and concurrent validity [36]. In order to receive an impression about the residents’ Quality of Life, the Quality of Life in Alzheimer’s disease (QOL-AD; person with dementia-version, [37]) and the Euroquol (EQ) 5D-3L scale [38] were used. The QOL-AD (Cronbach’s alpha: .68–.79; [39]) measures Quality of Life through 13 items on a 4-point Likert scale, ranging from 1 (poor) to 4 (excellent). In the first part of the EQ-5D-3L, the health status of the resident is assessed with five questions (i.e., agility/mobility, care for oneself, usual activity, pain, anxiety/depression), the possible answers range from Level 1 (no problem) to Level 3 (extreme problems). In the second part the resident has to assess his or her health status on a visual analogue scale, ranging from 0 (poor) to 100 (excellent). This scale demonstrated satisfactory reliability (ICCs’ range: .70–.94; [36]).

Individual feelings of physical pain were assessed by the VAS-Pain scale [40] and the Pain Assessment in Advanced Dementia (PAIN-AD) Scale [41]. The VAS-scale is a visual scale ranging from 0 (no pain) to 10 (severe pain) in which residents are asked to rate their pain (reliability: *r* = .71–.94; validity: *r* = 0.71–0.78 and 0.62–0.91; [36]). The PAIN-AD is applied to persons who are unable to communicate their pain due to dementia or cognitive impairment. There are five categories (breathing, negative vocalization, facial expression, body language, consolability), which can be rated from 0 to 2. The sum of these scores results in the total score ranging from 0 (no pain) to 10 (severe pain). The PAIN-AD demonstrated satisfactory reliability (ranging from .50 to .67) and validity coefficients (*r* = .76–.95) [41].

Finally, the short form of the Mini Nutritional Assessment (MNA) was used to assess the nutritional status [42]. A score greater than 11 in the MNA indicates an acceptable nutritional status whereas a score from 8 to 11 indicates a risk of malnutrition and a score lower than 8 indicates a state of malnutrition. The diagnostic accuracy for predicting undernutrition was shown to be 98.7% [38].

### Statistical analysis

Study participant characteristics and prevalence of symptoms were explored with descriptive statistical methods. As the assessed continuous variables were not normally distributed, univariate comparisons were made using the Wilcoxon rank sum test. For binary variables, the Chi^2^ test with Yates’ correction for continuity for 2 × 2 contingency tables was used. However, for easier interpretability, we present means and standard deviation (if appropriate) within the text and the tables. Statistical analysis was conducted using R (Version 3.3.1; [43]). In order to avoid type 1 errors when performing multiple tests, we adjusted the significance level by the number of tests (Bonferroni correction). Differences between countries can be seen as being statistically significant when *p*-values were < .001 (instead of the more common α = .05). Missing data were not replaced except, in the MNA, if the item measuring the body-mass index (and the calf circumference) was missing, it was replaced by the median of all other existing observations (this affected 18.8% of all cases). With regard to the EQ-5D-3L (Euroquol), we do not report a global analysis but compared each of the six items separately between the countries. Also for the KATZ Index, the analysis was item-based. The staff/residents ratio was computed separately for each nursing home by dividing the sum of the full-time-equivalent of all health care professionals by the sum of residents. For each country, a mean of this staff/residents ratio was computed across nursing homes. In Austria, care staff included nurses and healthcare assistants. In the Czech Republic, in contrast to Austria, also social workers are part of the care team. However, as social workers in the Czech Republic do not perform direct care tasks and mainly work with the family and in order to make the staff/residents ratio better comparable across countries, we computed the ratio for the Czech Republic excluding the social workers.

## Results

### Sample

In total, 702 residents in eight Austrian and 964 residents in 14 participating Czech nursing homes were invited via the management of the nursing homes to take part in the study. In Austria, 571 residents (response rate: 81.3%) and in the Czech Republic 514 residents (response rate: 53.3%) agreed to participate and satisfied the inclusion criteria. Reasons for non-participation were resident refusing to participate, no consent from the family care giver or the legal representative, acute illness or process of dying and person hospitalized during study process. Most residents in both countries had the nationality of the respective country and had German or Czech as first languages (see Table 1).

**Table 1:** Sociodemographic data and dementia prevalence from the medical chart review

Country	AUT			CZ			*p*-value
Statistical values	*N*	Mean (±*SD*)^a^	95% CI	*N*	Mean (±*SD*)^a^	95% CI	
Sociodemographic data							
Female % (*n*)	571	73.4% (419)	69.8–77.0	514	77.8% (400)	74.2–81.4	.10^b^
Nationality % (*n*)	571	98.6% AUT (563)	97.6–99.6	496	96.4% CZ (478)	94.7–98.0	
Native language % (*n*)	571	98.3% German (561)	97.2–99.3	493	97.0% Czech (478)	95.4–98.5	
Age (yrs.)	571	84.4 ± 8.3 (Range: 50–102)	83.7–85.1	485	84.6 ± 7.5 (Range: 53–102)	83.9–85.3	.998^c^
Stay in nursing home (yrs.)	571	3.4 ± 4.5 (Range: 0–65)	3.0–3.8	467	3.1 ± 3.6 (Range: 0–22)	2.8–3.4	.47^c^
Diagnosis of dementia							
Dementia in chart % (*n*)	571	58.8% (336)	54.8–62.9	349	55.0% (192)	49.8–60.2	.28^b^
Alzheimer’s disease % (*n*)		34.5% (116)			37.5% (72)		
Vascular dementia % (*n*)		10.4% (35)			20.8% (40)		
Other (or not specified, CZ) % (*n*)		55.1% (185)			41.7% (80)		

^a^Data represent mean (*M*) and standard deviation (*SD*) if not otherwise stated

^b^Pearson’s Chi-squared test

^c^Wilcoxon rank sum Test

### Environmental features of the nursing homes in the two countries

Five out of the eight selected Austrian nursing homes were state owned; two were municipal, one home was church-owned. In the Czech Republic, residents of 14 different nursing homes were assessed. Seven of the 14 nursing homes were publicly owned (municipal), five were non-profit (NGO or church-owned) and two were for-profit nursing homes (i.e., run by private persons or companies). The number of residents ranged from 48 to 125 residents in the Austrian nursing homes (702 in total) and from 8 to 260 in those of the Czech Republic (964 in total). In Austria, five of the directors of the nursing homes were female, three directors were male. They were on average 45.1 years old (*SD* = 9.8) and 14.4 years (*SD* = 14.1) in service. Two of the directors were trained nurses; all others had completed training in health−/social- or administrative management. Five out of eight had in addition a special training on directing a nursing home. In the Czech Republic, 12 of the 14 nursing home directors were female. No further information was available on the qualification of the directors in the Czech Republic. In an interview with the nursing home managers, also the care concepts used in the nursing homes were investigated. In Austria, the managers reported person-centred concepts (6 homes), and medically oriented care concepts (2 homes). Additionally, 2 homes reported that they use validation communication methods. In the Czech Republic, most administrators reported person-centred concepts. No written concepts were present in either country.

With regard to the nursing home facilities, the portion of single bedrooms was higher in Austria (93.7%) than in the Czech Republic (25.3%). 57.2% of all bed rooms in the Czech Republic were double rooms, in Austria only 6.3% of residents lived in a double room. 17.5% of residents in the Czech Republic were living in multi-bed rooms (Austria: none). In seven out of eight (Austria) and 12 out of 14 nursing homes (Czech Republic), residents can, at least to some extent, bring their own furniture. All nursing homes investigated reported various social activities (e.g. arts and crafts, ceramics, movement therapy, physiotherapy, dance, music, reminiscence). Austrian nursing homes reported 3 to 11 different social activities per month (7.1 on average, *SD* = 2.3; average duration 30 to 120 min), with on average 16.9 (*SD* = 11.8) attendees per activity. In all of the Austrian nursing homes, mnemonic trainings and movement therapy were provided on at least a weekly basis, in one nursing home even on a daily basis (except weekends). Most of the nursing homes also provided other common activities (e.g., trips, celebrating mass). In the Czech Republic no detailed data about the activities were provided. In Austria, the staff/residents ratio was 0.45 (0.41 to 0.46 per nursing home), in the Czech Republic 0.47 (0.26 to 1.05 per nursing home).

### Sociodemographic description of the DEMDATA sample

In Table 1, the sociodemographic features of the DEMDATA sample are summarized. The majority of residents were female (AUT: 73.4%, CZ: 77.8%). The mean residents’ age was 84.4 years (*SD* = 8.33; Austria) and 84.6 years (*SD* = 7.51, Czech Republic), respectively, and did not differ across countries. On average, residents lived in the respective nursing home for 3.4 years (*SD* = 4.5) in Austria and 3.1 years (*SD* = 3.6) in the Czech Republic.

### Dementia diagnosis and dementia prevalence

In the Austrian sample, 58.8% of the residents (336 of 571 persons) had a dementia diagnosis in their medical chart (see Table 1). Within these cases, 34.5% (*n* = 116) of the residents had Alzheimer’s disease, 10.4% (*n* = 35) had vascular dementia and 55.1% (*n* = 185) had another form of dementia (e.g. Parkinson’s disease, Alcohol induced dementia). In the Czech Republic, researchers had access to only 349 out of 514 medical charts for legal reasons. From these 349 residents, 55.0% (*n* = 192) had a dementia diagnosis in their medical chart. 37.5% (*n* = 72) suffered from Alzheimer’s disease, 20.8% (*n* = 40) had vascular dementia and 41.7% (*n* = 80) had either another dementia or the type of dementia was not specified. Prevalence of dementia in the Czech sample based on the medical chart information did not differ from the Austrian sample.

**Fig. 1:**
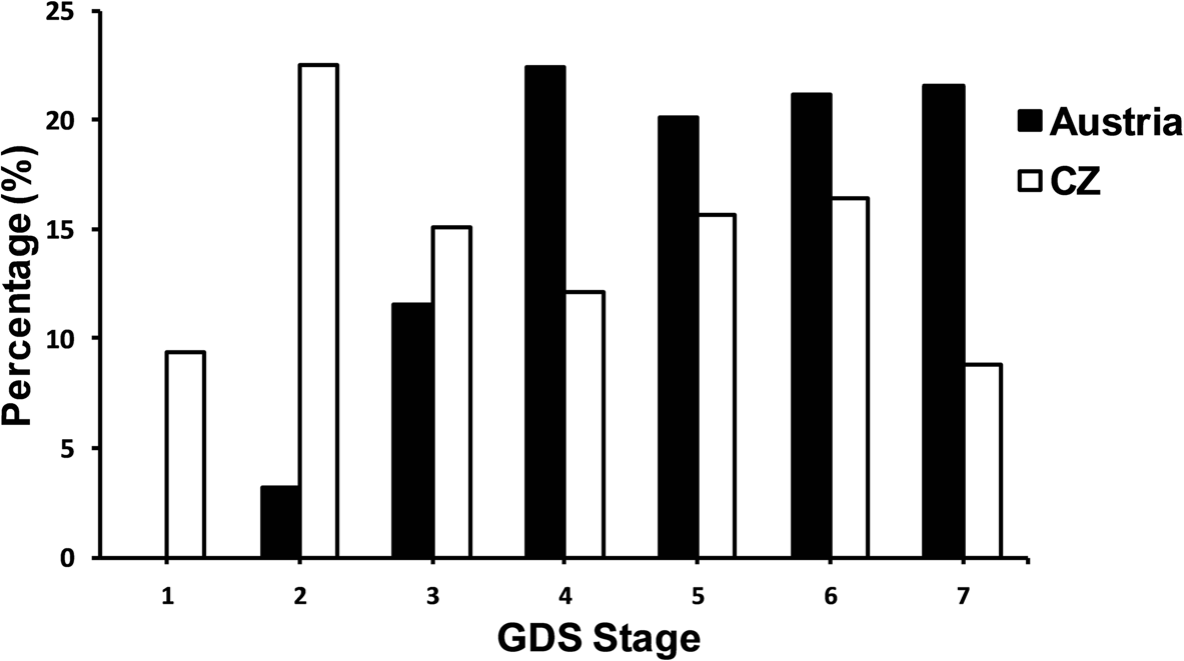
Percentage of residents in each GDS level for each country

However, findings from the Global Deterioration Scale (GDS) show differences between the countries, *p* < .001, see Table 2. In particular, the direct psychological screening revealed that 85.2% of the Austrian study participants (*n* = 479 out of 562) had a GDS score between 4 and 7, suggesting dementia stages from mild to very severe dementia (see also Fig. 1). The prevalence of dementia in the Austrian nursing homes based on the direct assessment was therefore 85.2%. In contrast, the assessed prevalence for the Czech Republic was 53.0%; i.e., 271 out of 511 tested residents had a GDS score between 4 and 7.

**Table 2:** Psychometric tests and clinical scale results from the DEMDATA sample

Country	AUT			CZ			*p*-value
Statistical values	*N*	Mean (±*SD*)^a^	95% CI	*N*	Mean (±*SD*)^a^	95% CI	
Cognition							
Global Deterioration Scale (GDS)	562	5.1 ± 1.4 (Range: 2–7)	5.0–5.2	511	3.9 ± 1.9 (Range: 1–7)	3.7–4.1	<.001^b^
GDS 1–3% (*n*)		14.8% (83)			47.0% (240)		
GDS 4–7% (*n*)		85.2% (479)			53.0% (271)		
MMSE	557	14.3 ± 9.4 (Range: 0–30)	13.0–15.1	489	17.7 ± 9.5 (Range: 0–30)	16.9–18.5	<.001^b^
Clock drawing test	552	4.9 ± 1.6 (Range: 1–6)	4.8–5.0	388	3.7 ± 1.8 (Range: 1–6)	3.5–3.9	<.001^b^
BCRS	564	4.9 ± 0.4 (Range: 1.3–7.0)	4.9–4.9	506	3.6 ± 0.9 (Range: 0.0–7.0)	3.5–3.7	<.001^b^
Behaviour							
BEHAVE-AD-FW	571	15.0 ± 19.9 (Range: 0–149)	13.4–16.6	513	7.1 ± 12.2 (Range: 0–79)	6.0–8.2	<.001^b^
Behavioural symptom, any % (*n*)		80.6% (460)	77.3–83.0		56.7% (291)	52.4–61.0	
E-BEHAVE-AD	569	0.8 ± 1.4 (Range: 0–10)	0.7–0.9	510	0.8 ± 1.6 (Range: 0–13)	0.7–0.9	.52^b^

^a^Data represent mean (*M*) and standard deviation (*SD*) if not otherwise stated

^b^Wilcoxon rank sum test

Moreover, 69.7% (159 out of 228) of the Austrian residents with no dementia in their medical chart showed significant signs of cognitive impairment (i.e., GDS stages 4 to 7), suggesting dementia. In addition, 4.2% (14 out of 334) had a dementia diagnosis in their chart but did not show signs of cognitive impairment (i.e., had a GDS stage 1 to 3). In the Czech Republic, 30.6% of residents (48 out of 157) with no dementia in the medical charts received a GDS stage between 4 and 7 and 15.7% of residents (30 out of 191) with a dementia diagnosis in their medical chart received a GDS score of 1 to 3, indicating no signs of cognitive impairment upon direct psychological testing.

### Cognitive test results

In the cognitive tests (see Table 2), residents of the Austrian nursing homes showed consistently and statistically significant lower mean scores than the residents of the homes in the Czech Republic, all *p*s <. 001.

### Behavioural problems

With regard to the BEHAVE-AD-FW 80.0% (*n* = 458) of Austrian residents and 56.7% (*n* = 291) of the Czech residents showed at least one behavioural symptom (see Table 2). In addition, the BEHAVE-score was higher in the Austrian than in the Czech sample, indicating significantly more behavioural problems in the Austrian sample, *p* < .001. Also one of the items of the EQ-5D-3L in which residents are asked about problems with regard to anxiety and depression, the Chi-squared test revealed a significant finding, *p* < .001. In the direct assessment of behavioural symptoms using the E-BEHAVE-AD (direct assessment), no difference between the countries was found however.

### Functioning

According to the EQ-5D-3L, 63.3% of the Austrian and 48.7% of the residents in the Czech Republic were restricted in their self-care. 36.7% (168 out of 458) Austrian and 51.3% of the Czech residents (234 out of 456) reported no problems with caring from themselves (see Table 3). The difference between countries was statistically significant, *p* < .001. With regard to “usual activity” (instrumental activities of daily living, IADL), 44.8% (205 out of 458) of the Austrian sample reported mild to severe problems; 55.2% (*n* = 253) no problems. This finding did not differ significantly from the finding of the Czech sample. Here, 37.7% (171 out of 454) reported mild to severe problems; 62.3% (*n* = 283) reported no problems. Furthermore, impairments in activities of daily living assessed with the Functional Assessment Staging (FAST) revealed an average score of 5.8 for Austria (*SD* = 1.4) and 4.1 (*SD* = 1.9) for the Czech Republic. In particular, 91.8% (524 out of 571) of the Austrian residents had a FAST-score greater than 3, indicating significant impairment, whereas in the Czech Republic, only 58.9% (299 out of 508) had a score greater than 3, *p* < .001. When analysing each item of the KATZ Index independently, we found only a significant difference for assistance in dressing, *p* < .001, but not in the other items (see Table 3).

**Table 3:** Functional measures and health outcome

	AUT			CZ			
Statistical values	*N*	Mean (±*SD*)^a^	95% CI	*N*	Mean (±*SD*)^a^	95% CI	*p*-value
FAST	571	5.8 ± 1.4 (Range: 1.0–8.0)	5.7–5.9	508	4.1 ± 1.9 (Range: 1.0–7.0)	3.9–4.3	
Fast (1–3) % (*n*)		8.2% (47)			41.1% (209)		<.001^b^
Fast (≥ 4) % (*n*)		91.8% (524)			58.9% (299)		
Katz-Index (need for assistance %, *n*)							
Bathing	565	78.4% (443)	75.0–81.8	509	75.4% (384)	71.7–79.2	.28^b^
Dressing	563	69.4% (391)	65.6–73.3	509	57.8% (294)	53.5–62.1	<.001^b^
Toileting	562	53.7% (302)	49.6–57.9	508	53.0% (269)	48.6–57.3	.84^b^
Transferring	564	40.4% (228)	36.4–44.5	509	45.0% (229)	40.7–49.3	.15^b^
Continence	565	69.6% (393)	65.8–73.4	509	71.7% (365)	67.8–75.6	.48^b^
Feeding	561	21.2% (119)	17.8–24.6	508	21.3% (108)	17.7–24.8	1.0^b^
EuroQol (EQ-5D-3L)							
Agility/Mobility % (*n*)	461	No problems: 21.7% (100) Mild to severe problems: 78.3% (361)	Mild to severe problems: 74.5–82.1	457	No problems: 25.6% (117) Mild to severe problems: 74.4% (340)	Mild to severe problems: 70.4–78.4	.19^b^
Care for oneself (ADL) % (*n*)	458	No problems: 36.7% (168) Mild to severe problems: 63.3% (290)	Mild to severe problems: 58.9–67.7	456	No problems: 51.3% (234) Mild to severe problems: 48.7% (222)	Mild to severe problems: 44.1–53.3	<.001^b^
Usual activity (IADL) % (*n*)	458	No problems: 55.2% (253) Mild to severe problems: 44.8% (205)	Mild to severe problems: 40.2–49.3	454	No problems: 62.3% (283) Mild to severe problems: 37.7% (171)	Mild to severe problems: 33.2–42.1	.035^b^
Pain % (*n*)	458	No problems: 55.2% (253) Mild to severe problems: 44.8% (205)	Mild to severe problems: 40.2–49.3	453	No problems: 48.6% (220) Mild to severe problems: 51.4% (233)	Mild to severe problems: 46.8–56.0	.051^b^
Anxiety/Depression % (*n*)	459	No problems: 61.0% (280) Mild to severe problems: 39.0% (179)	Mild to severe problems: 34.5–43.5	455	No problems: 73.0% (332) Mild to severe problems: 27.0% (123)	Mild to severe problems: 23.0–31.0	<.001^b^
State of health	414	62.0 ± 20.3	60.0–64.0	423	61.8 ± 21.6	59.7–63.7	.84^c^

^a^Data represent mean (*M*) and standard deviation (*SD*) if not otherwise stated

^b^Pearson’s Chi-squared test

^c^Wilcoxon rank sum test

### Mobility

 According to the sub-item “agility/mobility” of the EQ-5D-3L, the Austrian and the Czech sample did not differ with regard to their mobility (see Table 3). That is, 78.3% from the Austrian sample (*n* = 361) had problems with mobility or were immobile. 318 of these persons had some problems getting around and 43 were fullybedbound. One hundred persons from 463 assessed in the Austrian sample (21.7%) had no problems with mobility. In the Czech sample, 74.4% (*n* = 340) had problems with mobility. From these, 232 persons had some problems with mobility and 108 were bedbound. 117 persons from 457 assessed (25.6%) were fully mobile. The Timed-get-up-and-go test could be conducted on 43.8% of the Austrian sample (*n* = 250) and 51.4% of the Czech sample (*n* = 264) and demonstrated that the Czech sample was performing significantly better, *p* < .001 (see Table 4). 56.2% (*n* = 321) of the Austrian and 48.6% (*n* = 250) of the Czech residents could not perform the test because they were either bedridden, in a wheelchair, had a risk for falls or because of unknown reasons.

**Table 4:** Additional health related parameters

	AUT			CZ			
Statistical values	*N*	Mean (±*SD*)^a^	95% CI	*N*	Mean (±*SD*)^a^	95% CI	*p*-value
Timed-get-up and go test (seconds)	250	30.7 ± 17.9	28.5–32.9	264	26.7 ± 20.3	24.3–29.1	<.001^c^
VAS-Pain Scale	478	2.6 ± 3.1 (Range: 0–10)	2.3–2.9		not administered		
PAIN-AD	568	0.5 ± 1.2 (Range: 0–8)	0.4–0.6	508	0.6 ± 1.3 (Range: 0–8)	0.5–0.7	.35^c^
MNA, short form	546	9.8 ± 3.1 (Range: 0–14)	9.5–10.1	490	10.6 ± 2.9 (Range: 0–14)	10.3–10.9	<.001^c^
QOL-AD	435	32.9 ± 6.4 (Range: 3–51)	32.3–33.5		not administered		
Falls, past 6 months % (*n*)	571	38.9% (222)	34.9–42.9	474	22.4% (106)	18.6–26.1	<.001^b^
Hospital stays, past 6 months % (*n*)	571	30.3% (173)	26.5–34.1	447	16.6% (74)	13.1–20.0	<.001^b^

^a^Data represent mean (*M*) and standard deviation (*SD*) if not otherwise stated

^b^Pearson’s Chi-squared test

^c^Wilcoxon rank sum test

### Assessment of pain

In the EQ-5D-3L assessment (see Table 3) 253 out of 458 Austrian residents (55.2%) and 220 out of 453 Czech residents (48.6%) reported as having no pain whereas 205 Austrian residents (44.8%) and 233 Czech Residents (51.4%) complained about mild to severe pain. There was no statistical significance between the countries. The VAS-Scale, which was delivered only in Austria (see Table 4) reported values consistent with moderate pain, also showing a variation across residents for the Austrian sample (*M* = 2.6, *SD* = 3.1). The PAIN-AD, which was delivered in both countries showed similar findings for the Austrian sample (*M* = 0.5, *SD* = 1.2) and again revealed no statistical difference to the Czech residents (*M* = 0.6, *SD* = 1.3, see Table 4).

### Nutrition

Mean scores of the Mini Nutritional Assessment (MNA), were 9.8 (*SD* = 3.1) for the Austrian and 10.6 (*SD* = 2.9) for the Czech residents differed significantly between countries, *p* < .001 (see Table 4).

### Number of falls and hospital stays

In the Austrian sample, 38.9% (*n* = 222) of the residents had a fall within the past 6 months at least once and 30.3% (*n* = 173) had at least one hospital stay. In the Czech Republic, 22.4% (*n* = 106) residents fell at least once within the past 6 months and 16.6% (*n* = 74) had at least one hospital stay. In both cases, the difference was statistically significant, both *p*s < .001.

### Quality of life

In the sub-item “state of health” of the EQ-5D-3L, in which the residents indicate their state of health on a visual scale (thermometer) from 0 to 100 (0 being the worst possible state of health), the mean score was 62.0 (*SD* = 20.3) for Austria and 61.8 (*SD* = 21.6) for the Czech Republic. There was no statistical difference between the countries (see Table 3), indicating that health related quality of life was rated by most as “above average”. Finally, Quality of life of the residents (residents’ version; QOL-AD) was assessed in the Austrian sample only. In Austria, the average score of the QOL-AD was *M* = 32.9 (*SD* = 6.4, see Table 4).

## Discussion

This study aimed to explore the situation of persons living in nursing homes in Austria and the Czech Republic. 22 nursing homes participated in the study (8 in Austria, 14 in the Czech Republic). In Austria, the most common nursing home type is the state-administered nursing home. Therefore the proportion of state homes was the highest (five out of eight) and only one private home (a home owned by the church) and two nursing homes administered by the municipality needed to participate in order to guarantee a stratified sample. In the Czech Republic, the landscape of different environments providing care is more complex. Hence, a greater variety of environments was selected for participation. In some cases the access to medical information was denied (interpretation of the unclear legislation on health care provision in social institutions). This led to a response rate of only 53.3% in the Czech Republic. Therefore, the findings from the Czech Republic should be interpreted with caution. In Austria, no such problems were encountered. Residents of all stages of nursing home care were assessed without restrictions and response rate was high with 81.3%. A rather vague conceptualization of nursing home care was found in both countries. Most nursing home administrators described the concepts in some way as “person centred” and individualized care but no written concepts were available. In Austria, the majority of nursing home residents lived in single rooms. In the Czech Republic, the majority lived in double rooms. This difference is explained by state regulations in Austria that ask for single room care. Most nursing homes investigated in both countries allow the residents to bring - to some extent - their own furniture. To this date there is no clear evidence for both parameters (living in a single room and having your own furniture) as an important factor for the quality of life in nursing home residents. Studies point to the importance of personal possessions for the development of a sense of home [44]. Other investigations demonstrated that residents living in single rooms have undisturbed communication with staff and less conflicts with roommates [45]. The question remains unanswered whether the issues are relevant for persons in all stages of dementia alike. Social activities are, according to the information given by the nursing home administration, provided. The frequency and dementia stage specificity however requires further and systematic study. The resident/care team ratio was 0.45 in Austria and 0.47 in the Czech Republic. This ratio is higher in Austria than in previous years (2009: 0.37) but for both countries still not comparable to countries such as Norway (2009: 0.80, [9]).

In this study, dementia prevalence was not only investigated via chart review but also estimated via direct psychological assessment of cognitive functioning and ADL functioning. In Austria, the total estimation of dementia prevalence was 85.2%. This result comes close to the result of other studies presenting prevalence rates of 89.8% and 83.8%, respectively [11, 12]. Both studies compared chart review and direct testing, as we did in our study. In contrast, studies using proxy ratings report lower prevalence rates for cognitive impairment and dementia ([5]: 67%, [6]: 68%). In the Czech Republic, the total estimated prevalence rate for dementia was 55%. In the InterRAI study, a prevalence rate of 65.4% for the Czech Republic was reported [6]. The dementia prevalence rate for the Czech Republic found in the current study is also lower than results of previous studies [46]. This might be a result of a recent legislation on social services. Care allowance is usually higher for physical disability and therefore these persons are preferred before persons with dementia. In Austria, the access to nursing homes for potential residents has been limited by the introduction of seven care levels. Each care level is defined by hours of care per month and person. According to this policy, access to a nursing home is granted only upon care level 3, which means that a person requires more than 120 h of assistance per month. For levels less than 3, home care is supported by mobile services. This policy has been forced in the last years. It has to be noted further that in the Austrian sample, 70% of persons without a dementia diagnosis in their medical chart had, based on the assessment during the study, moderate to severe cognitive impairment. In the Czech sample, 30% were underdiagnosed (false negative) and 15% had a dementia diagnosis but no cognitive deficits upon testing (false positive). This finding points to a serious call for action for further research and improvement of medical services in this area.

In Austria 80%, and in the Czech Republic 56.7% of residents had behavioural problems. In comparison, Björk and colleagues used the NPI-NH and their prevalence rate was 92% [5]. In the SHELTER study that applied the InterRAI, a prevalence rate for behavioural problems of 27.5% was found. These substantial differences can most likely be explained by the different assessment methods applied in the different studies. The other reason could be that the samples assessed differ in their severity of cognitive impairment.

According to the FAST rating, which was achieved through a discussion of a clinician with a care person knowing the resident well, 91.8% of the Austrian sample and 58.9% of the Czech sample had a score of 4–7 which indicates significant decline in ADL functioning. This result indicates that functional impairment is even greater than cognitive impairment in the investigated sample. Taking the report of the residents on their own functioning capacity (EQ-5D-3L), the percentage of ADL impairment is lower since severely impaired persons can no longer provide reliable information.

There were no significant differences on the assessment of mobility between the two countries and the rate of impairment was very high (78.4% for Austria and 74.5% for the Czech Republic), providing some evidence that not all parameters are related to cognitive impairment only in our sample.

As for the prevalence of pain (44.8% in Austria, and 51.4% in the Czech Republic), our findings are comparable with those of other studies ([5]: 48%, [6]: 36%; and [47]: 43%). In both countries, the MNA indicates a risk of malnutrition. However, for this study we only analysed the mean scores but no prevalence rates. Further research in this area is desperately indicated and should provide a clear definition for malnutrition in order to provide practice relevant results [48].

The incidence of falls in our study differed in the two countries. In Austria, significantly more falls were reported. The percentage of persons having falls indicates the necessity to search for reasons and programs to reduce the incidence of falls [49].

There was no difference between the Quality of life ratings between the two countries and the mean scores rated by residents able to provide this information was “above average”. How this result, especially in the light of high prevalence rates for BPSD, ADL impairment and impaired cognition, should be interpreted, is difficult. On the one hand, studies demonstrated a high negative correlation between Quality of life and these parameters [39], on the other hand, cultural differences may be responsible for higher or lower ratings in Quality of life within European countries [50]. Additionally, studies demonstrated that Quality of life is rated differently by caregivers and by residents themselves. Caregiver’s rating resulted in lower scores indicating less quality of life [50, 51]. The psychological reasons for this discrepancy should be clarified in future studies.

### Data quality

The aim of this study was to provide quality data which can be further utilised by other researchers for national and international comparisons and as a basis for international studies. In order to achieve this, we developed a common study protocol, we made sure, testers were well-trained and that the data are entered and stored in a common data base. However some of the instruments provided difficulties in the research process and as a consequence were left out (for example the QOL-AD interview with residents) or not correctly scored (e.g., dental status). Some of the assessments could not be applied to all residents (e.g. due to intellectual disabilities, or if the residents refused the participation during the assessment).

### Importance of these findings for practice and recommendations for future research

In this study we found a high overall estimation of prevalence of cognitive deficit (suspected dementia), behavioural symptoms, pain and other health related factors. Especially the possible underestimation of dementia when comparing the medical charts of the residents with the findings from the direct testing indicates that diagnostic services need to be improved. It is to fear that persons having dementia but not receiving a medical diagnosis will not receive the medical and social attention they require. Collaborative international nursing home research has the advantage of exchanging experiences across national borders. Some structures in many countries are historically grown but not necessarily to the residents advantage. Therefore, the common reflection on future structures could speed up necessary progress. The development of common study protocols is bringing researchers onto a common ground of discussion.

We especially recommend establishing research assistants in the nursing homes recruited from within the care team of the nursing home during the research process. These research assistants are familiar with the special milieu of each house and, through respecting this, the burden on care team and the residents during the research process can be reduced. We suspect that even data quality is improved.

Agreeing on a common study protocol (study instruments) and a common data management was essential for this study. Data monitoring was made possible in an early phase of the study preventing missing data as much as possible. Piloting the protocol before the start of the study guarantees that the study instruments are accepted by all raters. However, in our study still some instruments were not performed in both countries (for example the QOL-AD). More severely impaired persons cannot be assessed with this instrument. Overall, the assessment of life quality in nursing home residents is still unresolved [42]. Common rater training could provide an additional quality element for future studies.

A profound analysis of country specific settings and policies is necessary in order to properly interpret the results. Visits of the researchers to experience the milieu of other countries could improve the understanding further. Most of our study instruments proved their usefulness in our study. However, country specific validations of the instruments are necessary to prevent measurement errors in future studies.

### Limitations of the study

This is a baseline evaluation of the residents’ data of the DEMDATA study. No predictors can be presented. Some instruments were difficult to be administered and some scales are also not internationally validated. This is a potential source of bias and should be considered in future studies. Some misunderstandings still occurred despite the common study protocol and the common data entry system. We could only estimate the prevalence of dementia, and dementia diagnosis should be confirmed by an interdisciplinary team involving radiologists, neurologists and geriatricians. We can also not exclude a bias in the selection of the study sample especially due to the limited access to potential study participants and medical records in the CZ Republic. In the Czech Republic, some wards were not made accessible to the researchers for unknown reasons. There is a possibility that these wards were mainly occupied with residents with dementia. Therefore the estimation of prevalence for the Czech Republic cannot be clarified within this study. Our study demonstrates that cross country comparisons are possible but also bring challenges.

## Conclusions

The aim of the current study was to provide information on the prevalence rates of different health parameters of residents living in nursing homes in Austrian and the Czech Republic. Our findings showed some similarities but also several differences between the countries and provide a good basis for the further development of research in this field. Understanding the true dimension of dementia prevalence and pain can stimulate the development of improved diagnostic services and methods of stage specific care and support methods for persons with dementia. Also considering the high prevalence rate of behavioural symptoms, one of the sources of burden for the care teams needs to be acted on in future research and practice. In each country efforts to improve nursing home care are undertaken. Therefore, learning from each other can be very fruitful if diagnostic processes are transparent and accessible for research.

## Abbreviations

ADL: Activities of daily living
AUT: Austria
BCRS: Brief cognitive rating scale
BEHAVE-AD-FW: Behavioral pathology in Alzheimer’s disease assessment scale /Frequency-Weighted
BPSD: Behavioural and psychological signs and symptoms of dementia
CZ: Czech Republic
E-BEHAVE-AD: Empirical behavioural pathology in Alzheimer’s disease assessment scale
EC: European community
Euroquol/EQ-5D-3L: Assessment of quality of life and health outcome
FAST: Functional assessment staging of Alzheimer disease
GDS: Global deterioration scale
IADL: Instrumental activities of daily living
ICC: Inter Class Correlation Coefficient
iLTC: Institutional long-term care
KATZ Index: ADL scale
M: Mean
MMSE: Mini mental state examination
MNA: Mini Nutritional Assessment
NGO: Non-governmental organization
OECD: Organization for economic co-operation and development
PAIN-AD: Pain assessment in advanced dementia-scale
QOL-AD: Quality of life in Alzheimer’s disease
SD: Standard deviation
VAS-pain scale: Visual analogue scale pain scale
χ^2^: Chi squared
